# Ex-Service Men and a Sussex Hospital

**Published:** 1921-03-26

**Authors:** 


### Ex-Service Men and a Sussex Hospital.

The ex-Service men of Chichester are to be congratulated on their decision to hand over to the Royal West Sussex Hospital the whole of the money—£450—which accrued to them from the United Services Fund. The ex-Service men wish it to be allocated, namely : £105 to endow a bed; £105 for massage treatment for wounded men; £105 for the $x$-ray department; £105 for special surgical cases; £30 to operating-theatre. It was originally intended, or suggested, that the money should be spent on a dinner.

